# Supplementary material for: MetaRibo-Seq measures translation in microbiomes
Source: Nat Commun. 2020 Jun 29;11:3268. doi: 10.1038/s41467-020-17081-z (PMC7324362; doi:10.1038/s41467-020-17081-z)
Supplement: Supplementary file 10 — Supplementary Data 7 [file 41467_2020_17081_MOESM10_ESM.zip › File2/Confidence_High_Taxonomy/159702_out.krona.html]

Javascript must be enabled to view this page.

members
magnitude
magnitudeUnassigned
count
unassigned
taxon
rank

159702\_out

5

5
2
superkingdom

phylum
976
5

class
200643
5

order
171549
5

815
1
family

1
816
genus

1
820
species

SRS017859\_contig\_number\_1925

family
4
171550

4
239759
genus

species

SRS015694\_contig\_number\_25461SRS049995\_contig\_number\_38927SRS062654\_contig\_number\_contig-100\_765.155725SRS098644\_contig\_number\_contig-100\_1964.272585
214856
4
